# Introduction to *Strongyloides stercoralis* Anatomy

**DOI:** 10.2478/jofnem-2024-0019

**Published:** 2024-06-09

**Authors:** Michelle L. Castelletto, Damia Akimori, Ruhi Patel, Nathan E. Schroeder, Elissa A. Hallem

**Affiliations:** Department of Microbiology, Immunology, and Molecular Genetics, University of California, Los Angeles, Los Angeles, CA, 90095; Molecular Biology Interdepartmental Ph.D. Program, University of California, Los Angeles, Los Angeles, CA, 90095; Department of Crop Sciences, University of Illinois at Urbana-Champaign, Urbana, IL 61801; Molecular Biology Institute, University of California, Los Angeles, Los Angeles, CA, 90095.

**Keywords:** *Strongyloides stercoralis*, skin-penetrating nematode, parasitic nematode, parasitic helminth, WormAtlas, anatomy, morphology

## Abstract

*Strongyloides stercoralis*, commonly known as the human threadworm, is a skin-penetrating gastrointestinal parasitic nematode that infects hundreds of millions of people worldwide. Like other *Strongyloides* species, *S. stercoralis* is capable of cycling through a single free-living generation. Although *S. stercoralis* and the free-living nematode *Caenorhabditis elegans* are evolutionarily distant, the free-living adults of *S. stercoralis* are similar enough in size and morphology to *C. elegans* adults that techniques for generating transgenics and knockouts in *C. elegans* have been successfully adapted for use in *S. stercoralis*. High-quality genomic and transcriptomic data are also available for *S. stercoralis*. Thus, one can use a burgeoning array of functional genomic tools in *S. stercoralis* to probe questions about parasitic nematode development, physiology, and behavior. Knowledge gained from *S. stercoralis* will inform studies of other parasitic nematodes such as hookworms that are not yet amenable to genetic manipulation. This review describes the basic anatomy of *S. stercoralis*.

The genus *Strongyloides* contains around fifty species of parasitic nematodes that infect hosts ranging from amphibians to humans, with most species having a narrow host range consisting of one or a few host species (Speare, 1989; Viney and Lok, 2015). The threadworm *Strongyloides stercoralis* infects humans, some nonhuman primates, and dogs; some infections have also been reported in cats (Viney, 2006; Wulcan et al., 2019). The first description of *S. stercoralis* infections in humans was in 1876, when soldiers returning from the region that is now Vietnam began suffering from severe diarrhea (Bavay, 1877). *S. stercoralis* infections in humans cause strongyloidiasis, a disease that has been termed a “disease of disadvantage” due to its prevalence in low-resource settings with poor sanitation infrastructure (Beknazarova et al., 2016). Current estimates place *S. stercoralis* infections at over 600 million people worldwide, primarily in tropical and subtropical regions (Buonfrate et al., 2020). However, due to diagnostic challenges such as low larval count in stool specimens and low parasite burden, the true incidence of *S. stercoralis* infections is likely underreported (Ericsson et al., 2001; Greaves et al., 2013; Czeresnia and Weiss, 2022).

*S. stercoralis* infects hosts as developmentally arrested, all-female, infective third-stage larvae (iL3s) (Fig. 1). The iL3s invade hosts through skin penetration (Schad, 1989; Viney, 2006; Lok, 2007; Viney and Lok, 2015; Viney, 2017; Page et al., 2018). Within the host, the iL3s develop into parasitic adults and ultimately establish an infection in the host small intestine. Some of the progeny of the parasitic females exit the host in feces, develop into iL3s in the environment, and subsequently infect a new host (Schad, 1989; Viney, 2006; Viney and Lok, 2015; Viney, 2017; Page et al., 2018). Other progeny instead develop into free-living adults in the environment, whose progeny develop into iL3s that infect a new host. An infection route unique to *S. stercoralis* can also occur when female progeny complete their life cycle within the same host, a phenomenon that is termed autoinfection (Schad, 1989; Viney and Lok, 2015; Czeresnia and Weiss, 2022) (Fig. 1).

**Figure 1:**
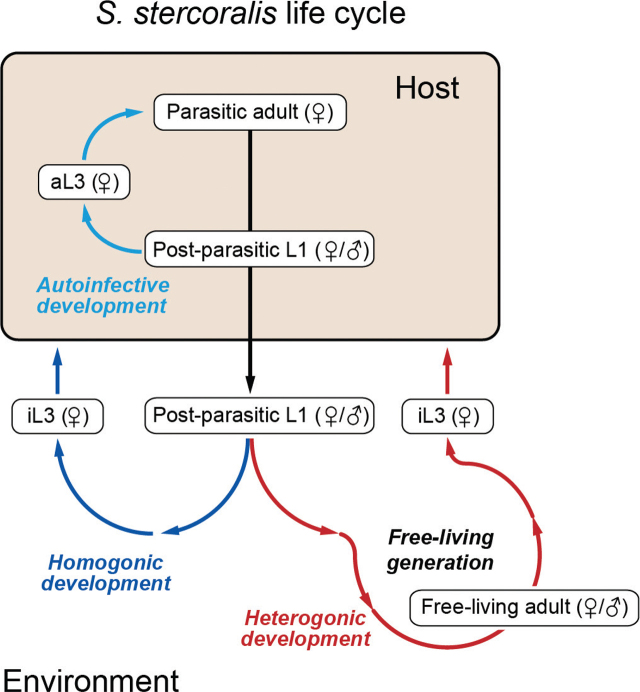
The life cycle of *Strongyloides stercoralis*. *S. stercoralis* post-parasitic larvae are a mix of males and females. An *S. stercoralis* post-parasitic first-stage larva (L1) can follow one of three developmental routes: homogonic (direct) development (female only), heterogonic (indirect) development (male or female), or autoinfective development (female only). A larva entering the heterogonic pathway develops into a free-living male or female adult. All progeny of the free-living adults become infective third-stage larvae (iL3s), which must infect a host to continue the life cycle. A larva entering the homogonic pathway develops directly into an iL3. Once inside a host, iL3s develop into parasitic adults. Finally, a larva that follows the autoinfective route develops into an autoinfective larva (aL3) and ultimately a parasitic adult inside the same host. All developmental pathways involve four larval stages (L1-L4); only the first and third larval stages are depicted. Image is adapted from Castelletto *et al.*, 2021 with permission (Castelletto and Hallem, 2021).

The severity of *S. stercoralis* infections among humans depends in part upon the health of the infected individual. *S. stercoralis* infections can be asymptomatic in healthy individuals, or they can cause gastrointestinal and pulmonary distress as well as eosinophilia (Nutman, 2017; Czeresnia and Weiss, 2022). Cycles of autoinfection in healthy patients can result in chronic strongyloidiasis, which can persist undetected for decades. In contrast, infections are often fatal in immunosuppressed individuals or individuals infected with certain viruses such as human T-lymphotropic virus 1 (HTLV-1) (Nutman, 2017; Czeresnia and Weiss, 2022). In these cases, the population of parasites engaging in the autoinfective cycle can increase rapidly, leading to hyperinfection and disseminated strongyloidiasis (Nutman, 2017; Czeresnia and Weiss, 2022; Herbert et al., 2022).

The development of *S. stercoralis* into a genetically tractable model organism has been bolstered by several aspects of its life cycle and morphology. *S. stercoralis* can be cultured in the lab by passaging it through dogs as a natural host or gerbils as a laboratory host (Nolan et al., 1993; Lok, 2007). In addition, *S. stercoralis* is capable of cycling through a single free-living generation (Schad, 1989) (Fig. 1). Free-living *S. stercoralis* adults are similar in size and morphology to *Caenorhabditis elegans* adults and can be easily recovered from laboratory host feces, making the free-living stages readily accessible for experimentation. Moreover, like *C. elegans* adults, *S. stercoralis* free-living adults have a syncytial gonad. Thus, exogenous DNA can be introduced into *S. stercoralis* free-living adults by intragonadal microinjection to generate transgenics and knockouts in their progeny using techniques adapted from *C. elegans* (Lok et al., 2017; Castelletto et al., 2020; Castelletto and Hallem, 2021; Mendez et al., 2022). Other human-parasitic nematodes lack a free-living generation, and as a result are less easily amenable to genetic manipulation. Thus, *S. stercoralis* has become a genetic model system for the study of human-parasitic nematode biology.

## *S. stercoralis* Life Cycle

*S. stercoralis* parasitic adult females colonize the mucosal layer of the host small intestine (Schad, 1989; Viney and Lok, 2015). They produce eggs via mitotic parthenogenesis and deposit them in the mucosal epithelium, usually in the crypts of Leiberkühn (Dionisio et al., 2000). The eggs hatch into male and female post-parasitic first-stage larvae, which then make their way into the host intestinal lumen (Little, 1966a). All male larvae pass into the environment with the feces and develop into free-living adults. The female post-parasitic larvae have three potential developmental routes: (i) They can pass into the environment with the feces and execute four molting cycles to become free-living adults. The free-living male and female adults reproduce sexually in the environment to produce all-female larvae that are restricted to becoming iL3s. (ii) They can pass into the environment and develop through two larval stages to become iL3s. (iii) They can develop through two larval stages within the host intestine into autoinfective third-stage larvae (aL3s), which subsequently develop to adulthood inside the same host (Schad, 1989; Viney and Lok, 2015) (Fig. 1). The ability to alternate between homogonic (direct to parasitism) and heterogonic (indirect to parasitism) life cycles is unique to nematodes in the genus *Strongyloides* and *Parastrongyloides*, and the ability to complete the life cycle within the same host through the generation of autoinfective larvae is thought to be unique to *S. stercoralis* (Grant et al., 2006; Nutman, 2017).

*S. stercoralis* iL3s are developmentally arrested third-stage larvae that resemble *C. elegans* dauer larvae (Schad, 1989; Viney et al., 2005; Ashton et al., 2007; Stoltzfus et al., 2012; Crook, 2014; Viney and Lok, 2015). The soil-dwelling iL3s navigate their environment to find and invade a host (Mendez et al., 2022). Once inside the host, the iL3s exit developmental arrest in a process called activation and migrate through the body to the lungs (Genta and Gomes, 1989). During this migration, the worms are thought to molt into fourth-stage larvae (Schad, 1989). They pass through the capillaries into the alveoli, causing tissue damage. Eventually, the larvae are coughed up and swallowed into the digestive system. Once the larvae have passed through the stomach and into the proximal small intestine, the duodenum, the larvae molt into reproductive parasitic adult females. Larvae undergoing autoinfective development penetrate through the host intestinal wall or perianal skin, then follow the same migratory route as iL3s, ending up as parasitic adults in the small intestine (Schad, 1989; Viney and Lok, 2015). In some cases, migrating larvae may bypass the lungs and arrive in the intestine via other routes (Schad et al., 1989; Mansfield et al., 1995).

## Anatomy

### Body Shape

#### Parasitic female

**Figure 2:**
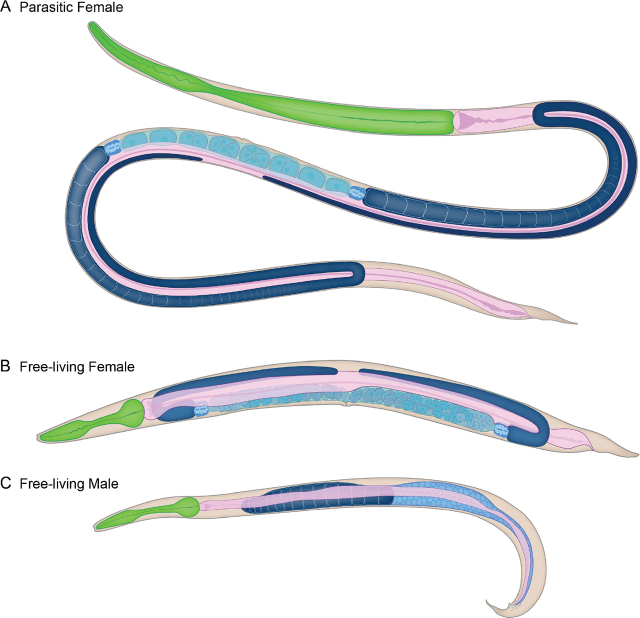
The adult stages of *S. stercoralis*. Schematics of an *S. stercoralis* (A) parasitic adult female; (B) free-living adult female; and (C) free-living adult male. Colored structures depict the pharynx (green), intestine (pink), and gonad (blue). Schematics are based on line drawings by Little, 1966 (Little, 1966a).

**Figure 3:**
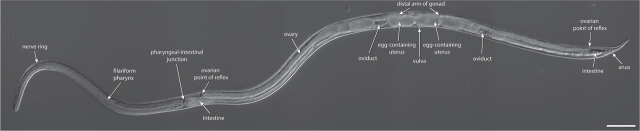
An *S. stercoralis* parasitic adult female. DIC image of an *S. stercoralis* young adult parasitic female. Scale bar is 100 µm. Image source: D. Akimori. DIC image was created using the Stitching plugin for ImageJ (Preibisch et al., 2009; Schindelin et al., 2012).

The *S. stercoralis* parasitic female resides in the host small intestine. An *S. stercoralis* parasitic female is long and slender, measuring approximately 2.4 mm in length and 37 µm in width (Schad, 1989; Lindo and Lee, 2001) (Figs. 2A, 3). *S. stercoralis* is sometimes called the threadworm because of its long, slender morphology (Lindo and Lee, 2001).

#### Free-living female

**Figure 4:**
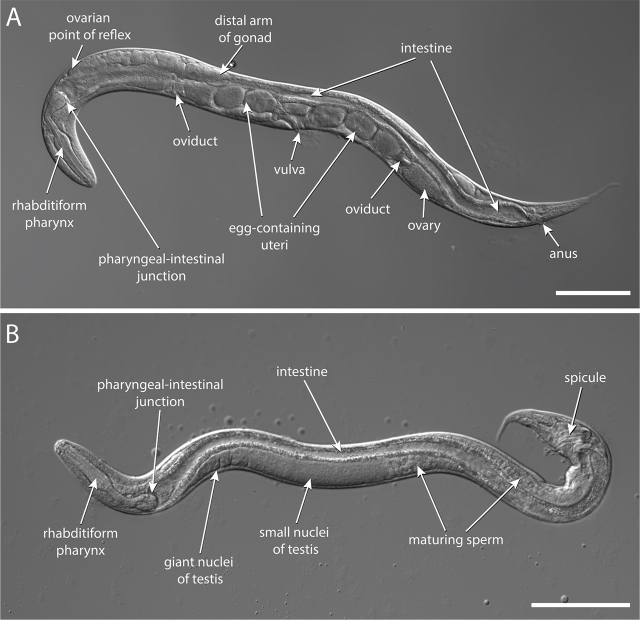
*S. stercoralis* free-living adults. (A) DIC image of an *S. stercoralis* free-living adult female; (B) DIC image of an *S. stercoralis* free-living adult male. Scale bars are 100 µm. Image source: D. Akimori. DIC images were created using the Stitching plugin for ImageJ (Preibisch et al., 2009; Schindelin et al., 2012).

*S. stercoralis* free-living females are approximately 1.1 mm long and 62 µm wide, with a conical pointed tail (Schad, 1989) (Figs. 2B, 4A). For comparison, a *C. elegans* adult hermaphrodite is approximately 1 mm in length and 80 µm in width (Palikaras and Tavernarakis, 2013). *S. stercoralis* free-living adults were estimated to have approximately 740 somatic nuclei, excluding somatic nuclei in the gonad and intestine (Hammond and Robinson, 1994a); this is roughly similar to the 959 somatic nuclei, including somatic nuclei in the gonad and intestine, found in *C. elegans* adult hermaphrodites (Sulston and Horvitz, 1977; Wood, 1988). The *S. stercoralis* cuticle and body wall are transparent, allowing easy visualization of internal structures.

#### Free-living male

The *S. stercoralis* free-living adult male is smaller than the free-living adult female, measuring approximately 0.9 mm in length and 43 µm in width (Schad, 1989; Lindo and Lee, 2001) (Figs. 2C, 4B). Their broad tail is slightly curved and contains a pair of spicules used for insemination of the females (Fig. 4B).

#### Rhabditiform larva

**Figure 5:**
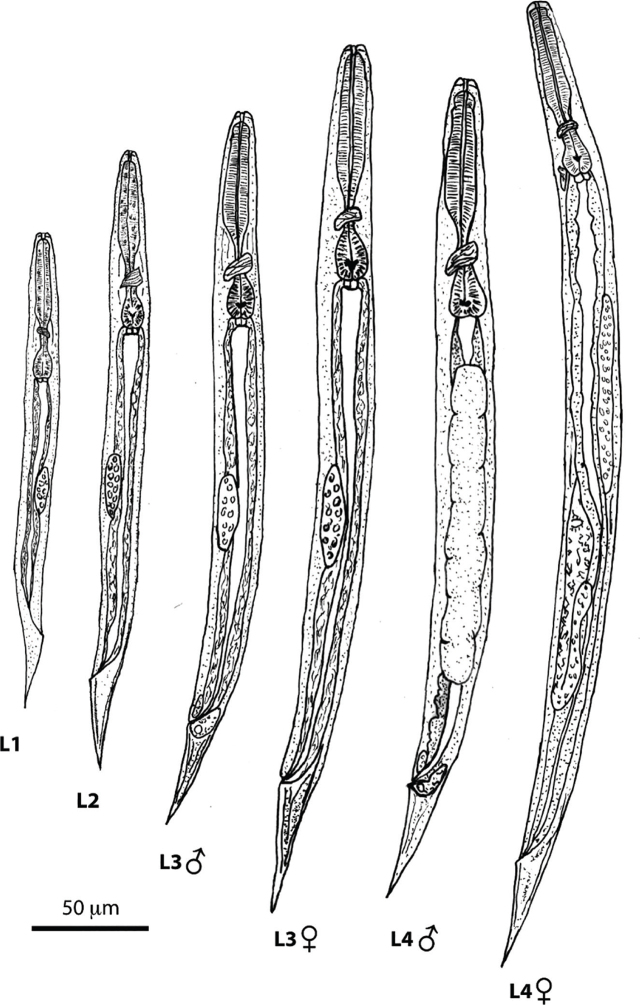
Line drawings of the rhabditiform larval stages of *S. stercoralis* undergoing free-living (heterogonic) development. Reproduced with permission from Buonfrate *et al.*, 2023 (Buonfrate et al., 2023).

**Figure 6:**
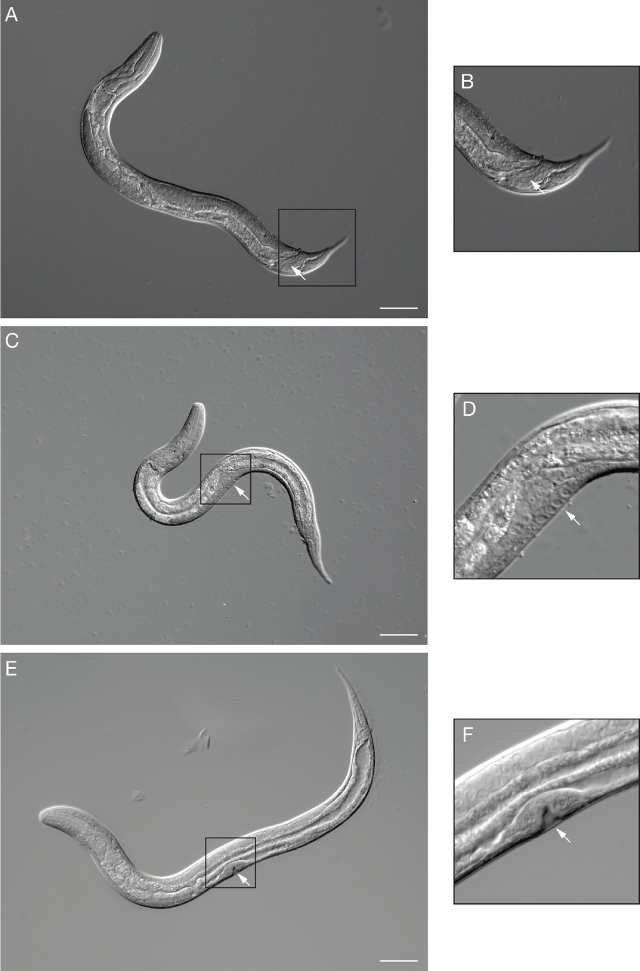
*S. stercoralis* free-living larvae. (A) DIC image of a male L4 larva. Box indicates the region enlarged in B. Arrow indicates the developing male copulatory structures. (B) Enlarged image of the tail of the male larva shown in A. Arrow indicates the developing male copulatory structures. (C) DIC image of a female L3 larva. Box indicates the region enlarged in D. Arrow indicates the developing vulva. (D) Enlarged image of the mid-body of the female larva shown in C. Arrow indicates the developing vulva. (E) DIC image of a female L4 larva. Box indicates the region enlarged in F. (F) Enlarged image of the mid-body of the L4 larva shown in E. Arrow indicates the distinctive invagination of the vulva that occurs at the L4 stage. For all images, scale bar is 50 µm. Image source: M. Castelletto.

Rhabditiform larvae include all first-stage (L1) and second-stage (L2) larvae, as well as third-stage (L3) and fourth-stage (L4) larvae that are destined to become free-living adults (Figs. 5, 6). Rhabditiform larvae consume bacteria and are identified by the presence of a rhabditiform pharynx. A hatchling first-stage larva in the intestine is 180–240 µm long and 14–15 µm wide. By the time they exit the host in feces, the L1s are approximately 250 µm long and 17 µm wide (Schad, 1989). L1 larvae are rounded on the anterior end, and their tail is slender and pointed. L2 and L3 larvae are larger than L1 larvae, with similar internal structures. However, the gonad increases in cell number and definition through each larval stage (Schad, 1989; Speare, 1989) (Figs. 5, 6). The reproductive structures of the free-living male can be identified in early larval stages (Figs. 6A,B).

L2s that will develop into iL3s have two morphological characteristics that foretell their different developmental track (Schad, 1989). First, in preparation for the increase in cell number in the longer intestine of the parasitic female, L2s fated to develop into iL3s have 22 intestinal cells and 40 nuclei instead of the 22 nuclei seen in L2s fated to develop into free-living adults. Second, the rhabditiform pharynx is slightly extended, and the posterior portion is more glandular than the anterior portion, predicting the altered pharyngeal structure of the iL3 and parasitic female (Schad, 1989).

#### Filariform larva

**Figure 7:**
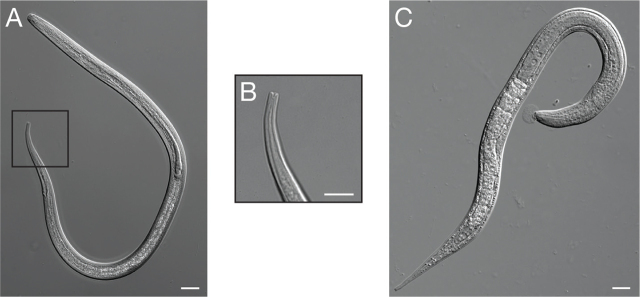
An *S. stercoralis* infective larva. (A) DIC image of an *S. stercoralis* iL3. Box shows the region enlarged in B. Scale bar is 20 µm. (B) Enlarged image of the iL3 tail, with its distinctive tetrafurcated tip. Scale bar is 10 µm. (C) DIC image of an activated *S. stercoralis* iL3 isolated from a gerbil intestine. Scale bar is 20 µm. Image source: M. Castelletto.

iL3s are also termed filariform larvae based on the structure of their pharynx. The morphology of the filariform larva is distinct from that of the other environmental stages, reflecting the anatomical changes required for host seeking and host invasion via skin penetration (Schad, 1989). iL3s are radially constricted, measuring approximately 560 µm in length but only approximately 16 µm in width. The filariform pharynx of the iL3s is approximately half of the body length, making this larva easy to identify under low magnification (Schad, 1989) (Fig. 7A). The tail of the *S. stercoralis* iL3 is tetrafurcated (Fig. 7B) (Nichols, 1956; Little, 1966a; Schad, 1989). The width of the iL3 increases substantially during the activation process as the pharynx expands and the larva resumes feeding (Fig. 7C).

#### Autoinfective larva

**Figure 8:**
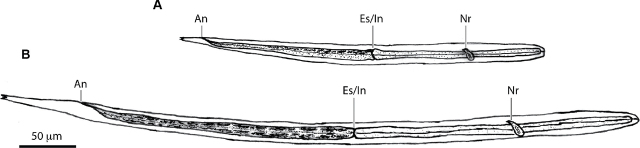
Comparison of an *S. stercoralis* autoinfective larva and an *S. stercoralis* iL3. (A) Line drawing of an autoinfective larva (aL3). (B) Line drawing of an iL3. An, anus; Es/In, esophageal-intestinal junction (also called the pharyngeal-intestinal junction); Nr, nerve ring. Adapted with permission from Buonfrate et al., 2023 (Buonfrate et al., 2023).

Third-stage autoinfective larvae (aL3s) are unique to *S. stercoralis*. These larvae develop within the same host as their parent parasitic female. They can be distinguished from iL3s based on their size (Fig. 8). The autoinfective larvae are shorter than filariform larvae, measuring approximately 270 µm in length and 11 µm in width (Humphreys and Hieger, 1979; Gocek et al., 1985; Kim et al., 2005). The aL3 pharynx is filariform and occupies approximately half of their length, like the pharynx of the iL3 (Kim et al., 2005). They also have the characteristic tetrafurcated tail of the filariform larvae (Schad et al., 1993; Kim et al., 2005).

### Organs and Tissues

#### Cuticle

The body wall of nematodes comprises the cuticle, the outermost layer of extracellular matrix components; the hypodermis, or epithelial tissue, which secretes the cuticle components; and the muscular layer (Page and Johnstone, 2007). The cuticle is acellular and is composed of collagens, cuticulin, and assorted surface proteins (Politz and Philipp, 1992; Page et al., 2014). The *S. stercoralis* cuticle has not been studied in detail, so knowledge of the cuticle of *Strongyloides* species comes primarily from studies of the rat-parasitic species *Strongyloides venezuelensis*.

#### Parasitic female

**Figure 9:**
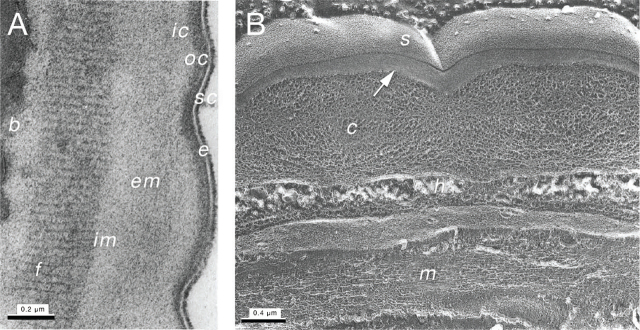
Cuticle of an *S. venezuelensis* parasitic female. (A) TEM image of the cuticle of an *S. venezuelensis* parasitic female. sc, surface coat; e, epicuticle layer; oc, outer cortical layer; ic, inner cortical layer; em, external medial layer; im, internal medial layer; f, fibrous layer; b, basal layer. (B) Freeze-fracture image of the body wall of an *S. venezuelensis* parasitic female. s, surface coat; c, cuticle; h, hypodermis; m, muscle. The arrow indicates intramembranous particles. Images in A and B are reproduced from Martinez and de Sousa, 1995 with permission (Martinez and de Souza, 1995).

The cuticle of the *S. venezuelensis* parasitic female measures approximately 1 µm thick and consists of seven main layers: the outermost epicuticle, outer cortical, inner cortical, external medial, internal medial, fibrous, and basal layers (Martinez and de Souza, 1995) (Figs. 9A,B). The *S. venezuelensis* epicuticle is covered in a surface coat that interacts directly with host tissues (Martinez and de Souza, 1995). The surface coat of parasitic nematodes may contribute to their ability to evade the host immune system (Blaxter et al., 1992). The cortical, medial, and basal layers of the cuticle have networks of thick and thin fibers with globular structures in an irregular arrangement (Fig. 9B). The composition of the fibrous layer is similar, except that the fibers are found in a more parallel orientation (Fig. 9A). The spaces between the collagenous fibers of the cuticle are thought to be filled with fluid. This open mesh of fibers likely allows nutrients and secretory products to diffuse across the cuticle while still providing the nematode with structural support (Martinez and de Souza, 1995).

#### Filariform larva

The molecular composition of the iL3 cuticle differs from that of other life stages such that it is more resistant to environmental stress (Martinez and de Souza, 1997). The basic structure of the *S. venezuelensis* iL3 cuticle has five layers: the epicuticle, cortical, medial, fibrous, and basal layers. The width of the cuticle is approximately 300 nm (Martinez and de Souza, 1997). Like the parasitic females, the iL3s have a surface coat on the exterior of the cuticle that is approximately 12 nm deep (Martinez and de Souza, 1997). The iL3s of the rat parasite *Strongyloides ratti* also have a surface coat that disappears once the iL3s have penetrated host skin (Grove et al., 1984; Grove et al., 1987). The purpose of this coat and its molecular composition are unclear.

**Figure 10:**
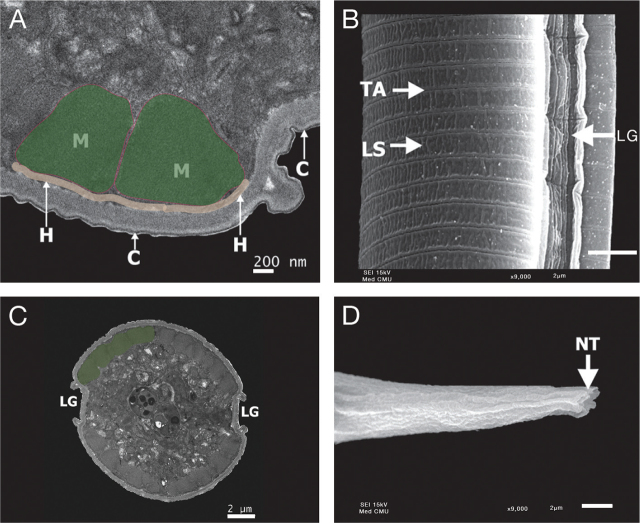
Cuticle of an *S. stercoralis* iL3. (A) TEM image of the cuticle of an *S. stercoralis* iL3. C, cuticle; H, hypodermis; M, muscle. Pseudo-coloring indicates muscle cells in green with pink outline and hypodermal layer in beige. (B) SEM image of the cuticle of an *S. stercoralis* iL3. TA, transverse annulations; LS, longitudinal striations; LG, longitudinal grooves. Scale bar is 2 µm. (C) TEM cross section of an *S. stercoralis* iL3. LG, longitudinal grooves. Pseudo-coloring indicates a subset of the muscle cells. (D) SEM image of the tail of an *S. stercoralis* iL3. NT, notched tail. The tail appears notched but is tetrafurcated (Nichols, 1956; Little, 1966a; Schad, 1989). Scale bar is 2 µm. Images are reproduced from Riyong et al., 2020 with permission (Riyong et al., 2020), with pseudo-color added in A and C.

*S. stercoralis* iL3s have two pairs of lateral alae running the length of the worm and extending slightly past the tip of the tail (Figs. 10A–D). These alae give the iL3 a characteristic tetrafurcated tail, which can be used to differentiate *S. stercoralis* iL3s from hookworm iL3s (Nichols, 1956; Little, 1966a; Schad, 1989; Lindo and Lee, 2001; Riyong et al., 2020) (Fig. 10D). The alae likely function to increase the stability of the worm during rapid movement, consistent with the increased motility seen at this life stage (Schad, 1989; Lindo and Lee, 2001).

#### Epithelial System

The hypodermis of nematodes lies under the cuticle and surrounds the body. It has several functions, including establishment of the body plan, production of cuticle components, and storage of nutrients (Altun and Hall, 2009c; Wolkow et al., 2021). In recent literature, the hypodermis is sometimes called the epidermis because it derives from the ectoderm (Altun and Hall, 2009c). In *C. elegans* and the beetle-associated nematode *Pristionchus pacificus*, the fusion of specialized epithelial cells during development results in multinucleate syncytia that form the hypodermis (Altun and Hall, 2009c; Schroeder, 2021). Thicker parts of the hypodermis, sometimes called the hypodermal cords, contain the hypodermal nuclei. The dorsal and ventral hypodermal cords house the dorsal and ventral nerve cords, respectively, while the lateral hypodermal cords serve as conduits for the excretory canals (Chisholm and Xu, 2012; Basyoni and Rizk, 2016). Little is known about the specifics of the *S. stercoralis* hypodermis.

#### Nervous System

**Figure 11:**
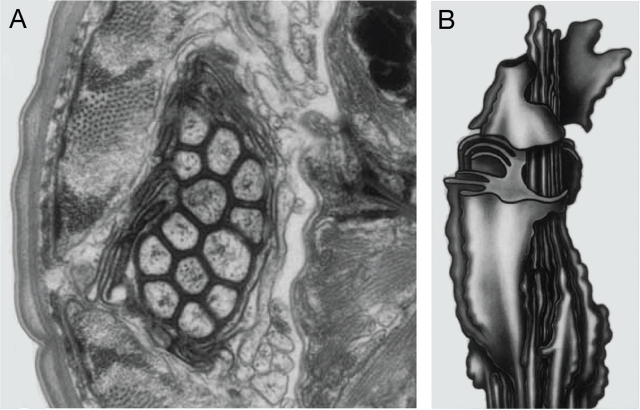
*S. stercoralis* amphid sensillum. (A) TEM image of one of the paired amphid sensilla of an *S. stercoralis* iL3. The amphid neurons (center) are connected to each other and to the sheath cell via tight junctions. Surrounding the amphid neurons are processes from the lamellar neuron, which was originally called the lamellar cell or ALD neuron (Ashton et al., 1995) but has since been identified as the AFD neuron (Bryant et al., 2022). (B) Diagram of the amphidial bundle, showing the lamellar processes of the AFD neuron surrounding the other amphidial neuron processes (left, front). Images are reproduced from Ashton et al., 1995 with permission (Ashton et al., 1995).

In general, the nervous system of *S. stercoralis* resembles that of *C. elegans* and other nematodes (Mendez et al., 2022). The structure of the anterior nervous system of *S. stercoralis* iL3s was determined by serial-section electron microscopy and found to resemble that of *C. elegans*, with several important differences (Ashton et al., 1995; Fine et al., 1997). Like *C. elegans, S. stercoralis* has paired amphid sensilla in the head that house many of the sensory neurons (Ashton et al., 1995) (Fig. 11). The amphid sensilla of the iL3s are open to the external environment, consistent with a role for sensory perception in host seeking and host invasion (Ashton et al., 1995). *S. stercoralis* has 13 pairs of amphid sensory neurons, in contrast to 12 pairs of amphid sensory neurons in *C. elegans* (Ashton et al., 1995). However, it remains unclear whether this is a true anatomical difference or a difference in nomenclature: the “extra” amphidial neuron in *S. stercoralis* has a short anterior process that terminates at the base of the amphidial channel and may correspond to the AUA neuron of *C. elegans* and *P. pacificus*, which is not considered a true amphidial neuron in these species (White et al., 1986; Ashton et al., 1995; Hong et al., 2019). The labial and cephalic sensilla of *S. stercoralis*, which are thought to be mechanosensory, are also morphologically similar to those of *C. elegans*, although there are some differences in the number of neurons per sensillum between the two species (Fine et al., 1997).

Although the cell body positions of the anterior neurons are roughly conserved between *C. elegans* and *S. stercoralis*, dendritic morphology is less well conserved (Ashton et al., 1995; Fine et al., 1997). For example, the AFD thermosensory neurons of *C. elegans* have a distinctive finger-like dendritic structure that is not present in *S. stercoralis* (Ashton et al., 1995; Goodman and Sengupta, 2018). There is only one amphid neuron in *S. stercoralis* that has a complex dendritic structure, and this neuron was originally termed the ALD neuron due to its lamellar dendritic structure (Ashton et al., 1995) (Fig. 11). A more recent genetic and functional characterization of this neuron identified it as the homolog of *C. elegans* AFD and the primary thermosensory neuron of *S. stercoralis*; this neuron has now been renamed AFD for consistency with *C. elegans* nomenclature (Bryant et al., 2022).

*S. stercoralis* has a clearly visible nerve ring in the head, which comprises densely packed neuronal processes (Ashton et al., 1995; Fine et al., 1997). In *C. elegans*, the nerve ring contains processes from more than half of the neurons in the body and is the most synapse-rich region of the nervous system (Altun, 2017). The nerve ring of *S. stercoralis* has not been studied in detail.

In *C. elegans*, the pharyngeal nervous system controls the activity of the pharynx and is isolated from the somatic nervous system. The pharyngeal nervous system of *S. stercoralis* has not been studied.

#### Muscle System

The body wall muscle of nematodes is separated from the hypodermal layer by a basal lamina (Basyoni and Rizk, 2016). The *S. stercoralis* musculature is platymyarian (the muscle fibers are adjacent and perpendicular to the hypodermis) and meromyarian (comprised of eight longitudinal muscle cells) (Little, 1966a; Basyoni and Rizk, 2016). The pharyngeal musculature, as well as the muscle cells involved in reproduction, male spicule movement, egg-laying, and defecation, have not been studied in *S. stercoralis*.

#### Excretory System

**Figure 12:**
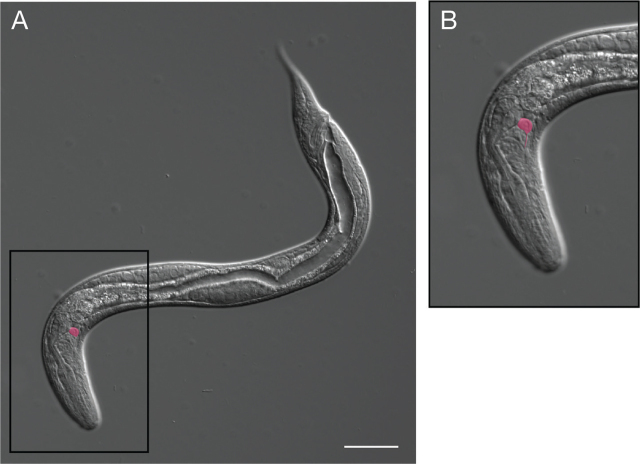
Excretory cell of *S. stercoralis*. (A) An *S. stercoralis* male L4 larva. The excretory cell is pseudo-colored. Scale bar is 50 µm. (B) Enlarged image of the head of the larva shown in A, with the excretory cell pseudo-colored. Image source: M. Castelletto.

The excretory system of nematodes has several functions, including maintenance of osmotic pressure, secretion of proteins into the environment, and elimination of waste (Sundaram and Buechner, 2016). The excretory system of *S. stercoralis* consists of two excretory canals running the length of the body along the lateral sides (Schad, 1989). A transverse duct connects the two canals to a single excretory cell, giving the *S. stercoralis* excretory system an H-shape like that of *C. elegans* (Schad, 1989,; Lindo and Lee, 2001; Sundaram and Buechner, 2016). The excretory cell is found posterior to the nerve ring, near the pharyngeal bulb (Nichols, 1956; Little, 1966a; Schad, 1989) (Fig. 12). A short canal ending in the excretory pore allows the contents collected by the excretory cell to be excreted into the environment (Lindo and Lee, 2001). The excretory pore opening is on the midventral line just posterior to the nerve ring, mid-pharynx (Schad, 1989; Lindo and Lee, 2001). The location of the excretory gland cells and neurons associated with the excretory system in *S. stercoralis* is unknown.

Studies of the excretory/secretory (ES) proteins of *S. stercoralis* have identified a wide variety of proteins that could be important for manipulation of the host immune response (Marcilla et al., 2010; Varatharajalu et al., 2011; Marcilla et al., 2012; Cuesta-Astroz et al., 2017; Hunt et al., 2017; Culma, 2021). However, where ES proteins are produced, how they are released from the worm and how they function remains unclear. The role of the excretory system in ES release is an open question and could be instrumental in understanding *S. stercoralis* host-parasite interactions.

#### Coelomocyte System

The coelomocyte system of *S. stercoralis* has not yet been characterized.

#### Alimentary System

As in other nematodes, the stoma, or mouth opening, connects to the pharynx, which pumps food into the intestine. The stoma, pharynx, and intestine of *S. stercoralis* differ significantly in parasitic females, free-living larval and adult stages, and iL3s.

#### Parasitic female

The stoma of the parasitic female can be used as an identifying feature for the different *Strongyloides* species (Little, 1966a, 1966b; Sato et al., 2008). In the case of *S. stercoralis*, the stoma is classified as angular and looks hexagonal when viewed *en face* (Little, 1966a). The stoma is surrounded by the circumoral elevation, a slightly elevated cuticle structure that consists of three paired lobes in *S. stercoralis* (Little, 1966a). *Strongyloides procyonis*, a parasite of raccoons, also has a hexagonal stoma shape (Little, 1966b; Sato et al., 2008). However, since *S*. *procyonis* does not infect humans, the hexagonal stoma can be used to differentiate *S. stercoralis* from the other human-infective species, *Strongyloides fuelleborni*, which has an X-shaped stoma (Little, 1966a).

The filariform (filament-shaped) pharynx of the *S. stercoralis* parasitic female extends up to one-third of the length of the body (Speare, 1989) (Fig. 3). The nerve ring encircles the pharynx, dividing it into an anterior one-fourth and posterior three-fourths (Schad, 1989). The expansion and contraction of the more muscular anterior portion of the pharynx together draws material into the stoma and the lumen of the pharynx (Lindo and Lee, 2001). The posterior region is glandular, with two subventral glands that deposit secretions into the pharyngeal lumen and one dorsal gland that deposits secretions near the mouth (Schad, 1989; Lindo and Lee, 2001). The exact functions and secretions of the pharyngeal gland cells are unclear, but it is possible these gland cells are a source of the ES products mentioned above. The pharynx connects to the intestine. The pharyngeal-intestinal junction is an easy-to-locate landmark a third of the way down the body (Fig. 3).

In nematodes, the intestine is responsible for the uptake of nutrients, disposal of waste products, and transport of signaling molecules (McGhee, 2007; Altun and Hall, 2009a). In general, the nematode intestine is composed of paired epithelial cells whose apical faces form the intestinal lumen and basal faces contact the body cavity (Altun and Hall, 2009a). Adherens junctions secure the epithelial cells together at the apical side (Altun and Hall, 2009a). The intestine is not anchored to the body wall; rather, it is anchored to the pharynx at the anterior end and the rectum at the posterior end (Altun and Hall, 2009a; Basyoni and Rizk, 2016). The intestine is lined with microvilli covered in a glycocalyx matrix (Altun and Hall, 2009a). The *S. stercoralis* parasitic female intestine is a thin tube with 40 uninucleate cells in two rows (Little, 1966a; Schad, 1989). It terminates near the tail in an anus that opens ventrally (Schad, 1989).

#### Free-living female

In free-living *S. stercoralis* females, the apical view of the stoma shows the oral opening to be dorsoventrally elongated, with two cephalic lobes extending above it (Little, 1966a). Each cephalic lobe has three papillae, small cuticle extensions that, based on knowledge of other nematodes and the lack of an opening to the external environment, are thought to be mechanosensory (Basyoni and Rizk, 2016). The paired amphid sensilla are located posterior to the papillae (Little, 1966a).

Free-living adults and other free-living life stages of *S. stercoralis* have a rhabditiform (rod-shaped) pharynx, in contrast to the filariform pharynx of the parasitic female and iL3. A rhabditiform pharynx is found in many bacterivorous nematodes, suggesting that *S. stercoralis* free-living adults feed on bacteria (Schad, 1989). In total, the rhabditiform pharynx of an *S. stercoralis* free-living adult is approximately 20% of the body length (Schad, 1989) (Fig. 4). The rhabditiform pharynx has three distinct regions: the procorpus, isthmus, and bulb (Schad, 1989). The anterior pharynx consists of the muscular procorpus, which attaches to the stoma. The isthmus connects the procorpus to the bulb containing the grinder. As in other nematodes, rhythmic contractions of the procorpus and the bulb draw bacteria into the pharyngeal lumen and pass it through the grinder into the intestine (Schad, 1989; Mango, 2007). Although the *S. stercoralis* grinder has not been studied in detail, the *C. elegans* grinder is composed of specialized cuticular structures that break up bacteria before it is released into the intestine (Altun and Hall, 2009b). The pharyngeal-intestinal valve is located approximately one-fifth of the way along the body in the adult females (Schad, 1989) (Fig. 4).

The intestine of the free-living life stages of *S. stercoralis* is composed of 22 uninucleate cells arranged in pairs, one dorsal and one ventral (Schad, 1989). It terminates near the tail, with the anal opening on the ventral midline (Schad, 1989). The intestinal cells are uninucleate but due to endoreplication–replication of the chromosomes without cell division–each nucleus may contain up to 16 times the haploid genomic material (16C) (Hammond and Robinson, 1994b). In contrast, the *C. elegans* intestine in adult hermaphrodites is composed of 20 cells with a total of 30–34 nuclei, with each nucleus containing up to 32C (McGhee, 2007; Altun and Hall, 2009a).

#### Free-living male

The stoma, rhabditiform pharynx, and intestine of the free-living male are as described for the free-living female (Schad, 1989).

#### Filariform larva

The iL3s have a compressed mouth and pharynx, reflecting the fact that iL3s are nonfeeding, developmentally arrested third-stage larvae similar to dauer larvae of nonparasitic nematodes (Viney et al., 2005; Ashton et al., 2007; Stoltzfus et al., 2012; Crook, 2014). The intestine of the *S. stercoralis* iL3 differs morphologically from the free-living intestine. The morphological differences are evident starting with L2s that are destined to develop into iL3s. The L2 intestine is composed of 22 cells in the same paired arrangement as the free-living adult intestine (Little, 1966a; Schad, 1989). However, there are 40 nuclei present, since all pairs except the most anterior and posterior pairs undergo nuclear division (Little, 1966a; Schad, 1989). At the iL3 stage, the intestine still has 22 cells with a total of 40 nuclei, but the intestine constricts, the lumen closes, and the intestinal cells fill with lipid droplets (Little, 1966a; Schad, 1989). After an iL3 enters an appropriate host, it initiates activation, the process by which it exits developmental arrest and resumes growth and feeding (Mendez et al., 2022). As part of this process, the pharynx and intestine expand, and the pharynx begins to pump (Fig. 7C).

#### Rhabditiform larva

The L1 pharynx occupies the first third of the body, while the intestine occupies the remaining two-thirds. The L1 intestine has the same basic structure as that of free-living males and females (Schad, 1989).

#### Reproductive System

*S. stercoralis* parasitic and free-living females are genetically identical; however, they are morphologically and functionally distinct life stages. Environmental, genetic, and host factors, including the immune status of the host, influence whether a female larva develops via the free-living or parasitic route (Viney et al., 2004; Viney and Lok, 2015). Parasitic females reproduce inside the host by parthenogenesis, generating a mix of male and female progeny (Hammond and Robinson, 1994a; Streit, 2008). Free-living females reproduce sexually, mating with free-living males to generate female progeny that will develop into iL3s (Yamada et al., 1991; Jaleta et al., 2017; Shao et al., 2017). Sex determination in *S. ratti* has been characterized as an XX/XO system with two pairs of autosomes and either a pair of X chromosomes in parasitic and free-living females or a single X chromosome in males (Harvey and Viney, 2001). *S. stercoralis* likely has the same XX/XO sex determination system, since it has the same number of chromosomes as *S. ratti* (Hammond and Robinson, 1994a).

#### Parasitic female

The reproductive system of the *S. stercoralis* parasitic female is amphidelphic, comprising two ovaries with opposed arms (Schad, 1989) (Figs. 2–3). The vulva opens on the ventral side and is located approximately two-thirds down the length of the body (Fig. 3). The egg-containing uteri extend anterior and posterior from the vulva opening and lead into ovaries that reflex, with the distal ends of the arms found opposite the vulva (Fig. 3). Importantly, the arms of the uteri are straight and do not spiral around the intestine. This characteristic is diagnostic of *S. stercoralis* and *S. ratti* (Schad, 1989); other *Strongyloides* species, including *S. fuelleborni*, have spiral ovaries (Little, 1966a). The uteri of parasitic females contain eggs in a single row (Schad, 1989) (Figs. 2,3).

#### Free-living female

**Figure 13:**
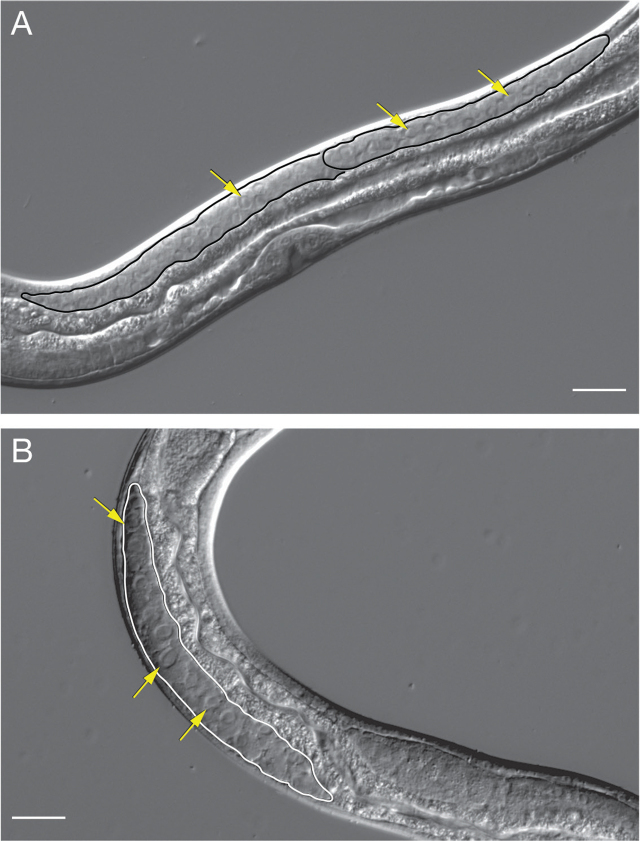
Syncytial gonads of *S. stercoralis* free-living larvae. Images show the syncytial gonads of *S. stercoralis* free-living (A) female and (B) male L4 larvae. Regions of the syncytial gonad are outlined; arrows point to selected nuclei in the syncytial gonad. Scale bar is 20 µm. A and B are enlarged versions of the images shown in Figures 6E and 6A, respectively. Image source: M. Castelletto.

Like the *S. stercoralis* parasitic female, the *S. stercoralis* free-living female has an amphidelphic reproductive system consisting of opposed gonad arms that recurve around the intestine (Schad, 1989). The vulva opening is located at approximately the ventral midpoint of the body (Fig. 4A). The uteri extend from the vulva opening in opposite directions and then curve back toward the center of the worm to end on the dorsal side of the body directly opposite the vulva (Schad, 1989) (Fig. 4A). The distal arm of the gonad is largely composed of giant polyploid nondividing nuclei (Hammond and Robinson, 1994a). Proximal to the giant nuclei is a region with small, highly compact nuclei, followed by a region with nuclei undergoing meiosis. Even more proximally, differentiated oocytes are found (Hammond and Robinson, 1994a). The distal gonad is syncytial and consists of germ cell nuclei in common cytoplasm surrounding a central rachis (Fig. 13A). The syncytial gonad permits the introduction of exogenous DNA by microinjection to generate transgenics or knockouts (Castelletto et al., 2020).

#### Free-living male

The *S. stercoralis* male reproductive system is morphologically simpler than that of the female–it consists of a single, straight tube that terminates at the cloaca (Schad, 1989) (Fig. 4B). The distal end of the testis is situated near the pharyngeal-intestinal junction and contains giant nuclei containing multiple copies of the genome (Hammond and Robinson, 1994a). The giant nuclei are followed by a region of small nuclei. The next visually distinct zone contains primary spermatocytes actively undergoing division to create spermatids, which will mature into sperm (Hammond and Robinson, 1994a). The copulatory organ of *S. stercoralis*, a pair of chitinous spicules, is found near the end of the tail and is extruded ventrally (Schad, 1989) (Fig. 4B). Situated in the cloaca, the spicules are connected to a second chitinous structure, the gubernaculum. Once a female has been encountered, the male coils its tail around the center of the female and uses the gubernaculum to both extend the spicules into the female vulva and retract them postcopulation (Schad, 1989). As in females, the syncytial nature of the distal gonad allows exogenous DNA to be introduced by microinjection (Shao et al., 2017) (Fig. 13B).

#### Rhabditiform larva

In the L1, the future reproductive system, or genital primordium, consists of about nine cells near the midbody. The number of cells in the gonad begins to expand around the time of the L2 molt. Thus, the cell count in the primordial gonad can be used to separate L1s from L2s (Lopez et al., 2000). A similar round of cell expansion occurs around the L3 molt (Lopez et al., 2000). Males and females are easily identifiable by the L3 stage based on the morphology of the tail and developing gonad structures (Figs. 6A–D). L3 and L4 females can be distinguished based on the development of the vulva. The L3 vulva comprises a group of cells in the mid-body on the ventral side, with the arms of the immature uteri extending posterior and anterior. The L4 vulva has a more distinct structure, including an invagination covered by the cuticle. The anterior and posterior uteri arms and ovaries are longer in the L4, and their structure is more defined (Figs. 6E–F).
